# Mapping QTL for Sex and Growth Traits in Salt-Tolerant Tilapia (*Oreochromis spp*. X *O*. *mossambicus*)

**DOI:** 10.1371/journal.pone.0166723

**Published:** 2016-11-21

**Authors:** Grace Lin, Elaine Chua, Laszlo Orban, Gen Hua Yue

**Affiliations:** 1 Temasek Life Sciences Laboratory, National University of Singapore, Singapore 117604, Singapore; 2 School of Biological Sciences, Nanyang Technological University, Singapore 637551, Singapore; 3 Department of Animal Sciences and Breeding, Georgikon Faculty, University of Pannonia, Deák Ferenc utca 16, H-8230 Keszthely, Hungary; 4 Centre for Comparative Genomics, Murdoch University, Murdoch, 6150 Australia; 5 Department of Biological Sciences, National University of Singapore, Singapore 117543, Singapore; James Cook University, AUSTRALIA

## Abstract

In aquaculture, growth and sex are economically important traits. To accelerate genetic improvement in increasing growth in salt-tolerant tilapia, we conducted QTL mapping for growth traits and sex with an F_2_ family, including 522 offspring and two parents. We used 144 polymorphic microsatellites evenly covering the genome of tilapia to genotype the family. QTL analyses were carried out using interval mapping for all individuals, males and females in the family, respectively. Using all individuals, three suggestive QTL for body weight, body length and body thickness respectively were detected in LG20, LG22 and LG12 and explained 2.4% to 3.1% of phenotypic variance (PV). When considering only males, five QTL for body weight were detected on five LGs, and explained 4.1 to 6.3% of PV. Using only females from the F_2_ family, three QTL for body weight were detected on LG1, LG6 and LG8, and explained 7.9–14.3% of PV. The QTL for body weight in males and females were located in different LGs, suggesting that in salt-tolerant tilapia, different set of genes ‘switches’ control the growth in males and females. QTL for sex were mapped on LG1 and LG22, indicating multigene sex determination in the salt-tolerant tilapia. This study provides new insights on the locations and effects of QTL for growth traits and sex, and sets the foundation for fine mapping for future marker-assisted selection for growth and sex in salt-tolerant tilapia aquaculture.

## Introduction

Increasing demands for fish to feed the ever-growing human population has highlighted the important role of aquaculture in fulfilling this need [1]. To ensure sustainability of the industry, selective breeding with genetically improved fish stocks is expected to lead to efficient utilization of tangible resources such as land space, feed, water and labor [2]. Growth is one of the most desired and economically important traits for aquaculture. Although traditional phenotype-based selections for growth are effective, they are labor-intensive, time consuming, and influenced by environmental factors [2]. Marker-assisted selection (MAS) is expected to increase the precision of selection and shorten the breeding cycles [3,4]. Mapping of quantitative trait locus (QTL) is an essential step towards MAS [3]. Despite the initial slow progress of QTL mapping in aquatic species compared to that in domestic animals, the advancement of new sequencing technologies and decreasing cost of genotyping have allowed for some catching up [5]. Till date, QTL mapping has been conducted for most important traits in over 20 aquaculture species [5,6], such as Asian seabass [7], in salt-tolerant tilapia [4] Japanese flounder [8], Nile tilapia [9], hybrid catfish [10] and Atlantic salmon [11]. These studies led to a better understanding of the locations and effects of QTL for important traits, and set up the foundation for MAS.

Tilapia, a common name of a group of cichlid fishes native to Africa is the second most cultured freshwater species group worldwide with a production of more than 5.3 million tons in more than 135 countries [12,13]. However, due to the limit of freshwater, extension of the Nile tilapia culture is limited. Mozambique tilapia (*Oreochromis mossambicus*) and its hybrids, including red tilapia, are representatives of euryhaline cichlids. Red tilapia are genetic mutants selected from tilapia species in the genus *Oreochromis* and their genetic heritages are not well documented as most strains of red tilapia have been crossed with unknown parentages including the Mozambique tilapia [14,15]. In the region, breeding through selection programs has stemmed from the Genetically Improved Farmed Tilapia (GIFT) program, increasing the growth performance of tilapia with every generation [16,17]. Mozambique tilapia and its hybrids are salt-tolerant, can be cultured in brackish water [18] and taste better than freshwater Nile tilapia [19], yielding them the potential to become an important marine foodfish species. However, salt-tolerant tilapia grows slowly when compared to the genetically improved Nile tilapia [20]. Despite the ongoing efforts to increase the growth performance of hybrid salt tolerant tilapias for culture, there has been mixed results due to the vague genetic background of the parental strains [21]. Fortunately, an increasing number of genomic resources are publicly available for tilapia, such as microsatellites [22], single nucleotide polymorphisms (SNPs) [23,24], genetic and physical maps [25,26,27,28], expressed sequence tags (ESTs) [29], whole genome assembly [30] as well as QTL for economical traits [4,31,32]. These resources provide the essential tools in MAS of tilapia. However, most of the previous studies were conducted mainly with Nile tilapia, limited studies on QTL mapping for important traits [25] in salt-tolerant tilapia were carried out.

In tilapia species, sex plays an important role in growth. In most tilapia species, including Nile and Mozambique tilapia, males grow much quicker than females [33]. However, the sex determination mechanism in tilapia is complicated. Sex is determined by genetic factors, environmental factors, social factors and their interactions [34]. In tilapia species, sex determination loci were mapped on LG1 [27,35], LG3 [35], LG23 [36,37] in different populations. In salt-tolerant tilapia, the sex determining locus has been mapped in LG22 [25].

The purpose of this study was to perform an initial genome scan for QTL associated with growth traits and sex in a F_2_ full sib family of salt-tolerant tilapia that was cultured in full seawater (i.e. 30 PPT seawater). We performed QTL analyses, and detected QTL for sex and growth traits on two and twelve linkage groups (LG), respectively. Majority of the QTL for body weight in males and females were located in different LGs, suggesting that in salt-tolerant tilapia, a different set of gene ‘switches’ control growth. This study provides us an overview on the locations and effects of QTL for growth traits and sex in salt-tolerant tilapia. It also establishes a starting point for fine mapping and future marker-assisted selection for growth in salt-tolerant tilapia aquaculture.

## Materials and Methods

### Ethics statement

All handling of tilapia fish in this study was conducted in accordance with the guidelines on the care and use of animals for scientific purposes set up by the Institutional Animal Care and Use Committee (IACUC) of the Temasek Life Sciences Laboratory (TLL), Singapore. The TLL’s IACUC has specially approved this study within the project “Breeding of Tilapia” (approval number TLL (F)-12-004).

### Mapping population and salinity acclimatization

One Mozambique tilapia male (from Genomar SEA, Singapore) and one red tilapia female (from a local farm in Singapore) of approximately 10 months old were randomly selected and used to generate an F_1_ family. One F_1_ male and one F_1_ female were selected from the F_1_ family to generate an F_2_ Family. The Mozambique tilapia male weighing 521g was randomly selected from a F2 generation that originated from F1 population of wild populations in South Africa. The red tilapia female, weighing 723 g, was a local genetically improved (quick growth and color) hybrid strain obtained from a local farm in Singapore. The F_2_ family used in our studies consists of 522 offspring individuals cultured in the fish facility of Temasek Life Sciences Laboratory. At 17 days post-hatch (dph) in 200 L tanks, the fingerlings were transited from freshwater to seawater over a period of one week by adding 30 PPT of filtered seawater at a flow-rate of 3.7 L/Hour and maintaining the ammonium levels of the water below 0.5 mg/L and pH at 8.0. During the period of transition, the fingerlings were fed to satiation three times daily with commercial feed containing 41.7% protein, 22.1% fat, 3.5% fiber, 1% calcium, 0.9% phosphorus, and 0.3% sodium (Biomar, Nersac, France). When the fingerlings were fully transitioned into 30 PPT seawater, they were transferred into 3-ton tanks using a flow-through system situated at the Marine Aquaculture Centre, Singapore and maintained on a strict feeding regime till harvest at 180 dph.

### Phenotyping of growth traits and analysis of correlation between traits

Body weight (BW), total length (TL) and body thickness (BT) were recorded at 180 dph for the 522 F_2_ individuals. BT was measured as the biggest width across the lateral line of the fish [38]. Dorsal fin clips of both parents and all offspring were sampled and preserved in absolute ethanol and stored in -20°C for DNA isolation with the method described earlier [39]. Sex was determined through manual observation of the gential pore. The trait data was recorded with binary code; male (trait value of 1) and female (trait value of 0). Fishes with unclear distinction of sex or under developed reproductive organs were also recorded. Pearson’s correlation coefficients between traits were calculated using statistical functions in Microsoft Excel.

### DNA isolation and genotyping of microsatellites

DNA of each fish was isolated with the method described earlier [39]. A total of 144 polymorphic microsatellite (MS) markers covering the 22 linkage groups (LG) of the tilapia genome near-evenly were selected from a published linkage map [25]. Detailed information about these markers is provided in S1 & S2 Tables. PCR amplification using fluorescently labeled primers [25] was carried out for each sample in a 25 μL reaction volume containing 10 ng genomic DNA, 1 x PCR buffer containing 1.5 mM MgCl_2_, 0.2 μM dNTPs, 0.2 μM forward and reverse primers, and 0.5 units of Taq DNA polymerase (Finnzymes, Espoo, Finland). The PCRs were performed in PTC-100 thermal cycler (MJ Research, CA, USA) under the following conditions: 2 min denaturation at 94°C; 35 cycles of 30 s at 94°C, 30 s at 55–60°C and 30 s at 72°C and a final extension at 72°C for 10 min. The PCR products were resolved on an ABI3730xl DNA Genetic Analyzer (Applied Biosystems, Foster City, USA). Fragment sizes were analyzed against the internal size standard of GeneScan-500 ROX (Applied Biosystems, Foster City, USA) using GeneMapper 4.1 (Applied Biosystems, Foster City, USA). Our genotyping system is able to differentiate size difference of one base pair among alleles. The genotypes were exported to an Excel table for further data analysis.

### Linkage map construction

Linkage maps were reconstructed using the JoinMap 4.0 software [40] for all offspring, male offspring, female offspring, as well as male and female offspring respectively. Genotyping data was formatted using the standard codes for “cross pollination” (CP) type progeny and was checked for inconsistencies with Mendelian inheritance and manually corrected for error. Samples with more than four missing genotypes were removed. The Kosambi mapping function was applied for the ‘best-fit’ map distance in the analysis and markers with LOD > 3.0 for segregation data were assigned to the same linkage group for all linkage maps. The sexing of the fishes were conducted with high stringency ensuring that only those fishes with a clear distinction of sex were used for the construction of the male and female linkage map. The number of individuals and markers used for constructing the all offspring, male offspring and female offspring linkage maps were N = 522, 125 markers; N = 235, 124 markers and N = 164, 123 markers, respectively. In addition, to map the sex determining loci, a linkage map was also constructed by using 399 F_2_ individuals with known sex and 125 DNA markers. All linkage groups were numbered in correspondence to the published map [25] based on selected markers. The maps were visualized using MapChart 2.1 software [41]. Total map length, average marker distance and female to male recombination ratio were calculated using statistical functions in Microsoft Excel.

### Mapping of QTL for growth traits

QTL mapping for growth traits was performed using MapQTL 5.0 software [42] for all offspring, male offspring and female offspring, respectively. Interval mapping (IM) analysis [43] based on maximum likelihood approach was used to identify the putative QTL at each position of the genome, where the genome was scanned at 1 cM intervals between loci and to associate markers with QTL. Cofactors were selected using the automatic cofactor selection analysis, and multiple-QTL mapping (MQM) [44] analysis was performed in order to remove the residual effects of other putative QTL located in the same linkage group and to increase the sensitivity of QTL detection.

Permutation test based on the empirical distribution values of randomizing trait values with respect to the genotypes was used to determine the significant Log of Odds (LOD) thresholds under null hypothesis of no segregating QTL [45]. The permutation test was performed with 10,000 re-iterations to determine the LOD threshold across single chromosomes as well as genome wide. QTL detected were deemed as suggestive if the LOD scores fell between 5% to 1% at the chromosome wide level, and significant if the LOD scores fell below 1% at chromosome wide or fell below 5% at genome wide level [46].

### Mapping QTL for sex

For QTL mapping, a linkage map was constructed using the 399 F_2_ individuals with known sex. The trait data was recorded with binary code; male (trait value of 1) and female (trait value of 0). Interval mapping (IM) [43] based on maximum likelihood approach was used to identify QTL for sex using MapQTL 5.0 software [42] as described above.

### Fine mapping of significant QTL for growth traits in male offspring

To fine map QTL for growth traits detected on LG 2 and 18, NCBI-blastn was performed against the assembled tilapia genome [30] using primer sequences of microsatellites located within the detected significant QTL for growth traits on LG 2 and 18. Genome sequences flanking these QTL were extracted and 18 additional microsatellites were identified. Primers for these additional 18 microsatellite markers (S2 Table) were designed using Primer 3. All 235 male offspring in the F_2_ family were genotyped. The male genetic map of LG 2 and 18 was reconstructed to include the additional markers and repeated QTL analysis was performed on a genome wide scale using IM as described above.

## Results

### Phenotypic values of growth traits and their correlations

Phenotypic data analyses of 522 fishes at 180 dph revealed that all three traits (BW, TL and BT) exhibited substantial levels of phenotypic variation (Table 1). The average body weight, total length and body thickness of all offspring was 270.2±70.8 g, 223.4±20.6 mm and 61.3±7.2 mm, respectively. The average body weight, total length and body thickness of the male offspring was 291.3±66.0 g, 231.6±17.7 mm and 63.5±6.9 mm, respectively. The average body weight, total length and body thickness of the female offspring was 239.2±63.8 g, 213.0±19.2 mm and 59.7±7.0 mm. Strong correlations (*r* = 0.93) was observed between body weight and total length (S3 Table). The correlation between body weight and body thickness and total length and body thickness were moderate (*r* = 0.7 and 0.69) (S3 Table). From the 522 F_2_ offspring at 180 dph, 235 were male and 164 were female, while for the remaining 123 individuals, the sex could not be determined.

**Table 1:** Phenotypic averages and standard deviations for growth traits for all, male and female offspring * recorded in 180 dph (days post hatch) in an F_2_ full-sib mapping family of salt-tolerant tilapia.

Trait values	All (n = 522)	Male (n = 235)	Female (n = 164)
BW (g)	270.2±70.8	291.3±66.0	239.2±63.8
BL (mm)	223.4±20.6	231.6±17.7	213.0±19.2
BT (mm)	61.3±7.2	63.5±6.9	59.7±7.0

BW: Body weight, BL: Body length, and BT: Body thickness.

*The male and female offspring in the population have been sexed manually with high stringency, fishes with unclear distinction of the sex has been removed.

### Linkage maps for all offspring, male offspring, female offspring as well as male and female offspring

Linkage maps for all offspring, only male, only female, as well as male and female offspring were constructed. The four linkage maps (S1, S2, S3 and S4 Figs) were in good agreement with the 22 linkage groups (LG) of the map published earlier [25]. Out of 144 markers used for mapping, 125, 124, 123 and 125 loci were anchored in the all offspring, only male, only female, and male and female offspring maps, respectively. The only male linkage map was firstly constructed with 124 markers, and later an additional 18 microsatellite markers (S2 Table) that were mapped on LG 2 and 18 (S2 Fig) to fine map QTL. The total map length, number of markers and average marker distance were 1170.6 cM, 125 and 11.4 cM; 1180.6 cM, 142 and 9.8 cM; 1162.1 cM, 123 and 11.5 cM and 1162.1 cM, 125 and 15.6 cM for all offspring, only male, only female, and male and female offspring maps, respectively (S4 Table).

### Comparison between male and female maps

The maps of male and female offspring contained 142 and 123 markers, and spanned 1180.8 and 1162.1 cM, respectively (S4 Table). These two maps shared 116 markers, and the total lengths of the linkage map based on shared markers were 1049.2 and 1051.3 cM in male and female offspring, respectively. The ratio of lengths of the common interval in female and male offspring was 1.00. Even though the length of the linkage map of female and male offspring were similar, the recombination ratios were significant on three linkage groups; 1.87, 0.67 and 0.67 on LG10, LG16, LG17 (S5 Table).

### Mapping QTL for sex

**Fig 1:**
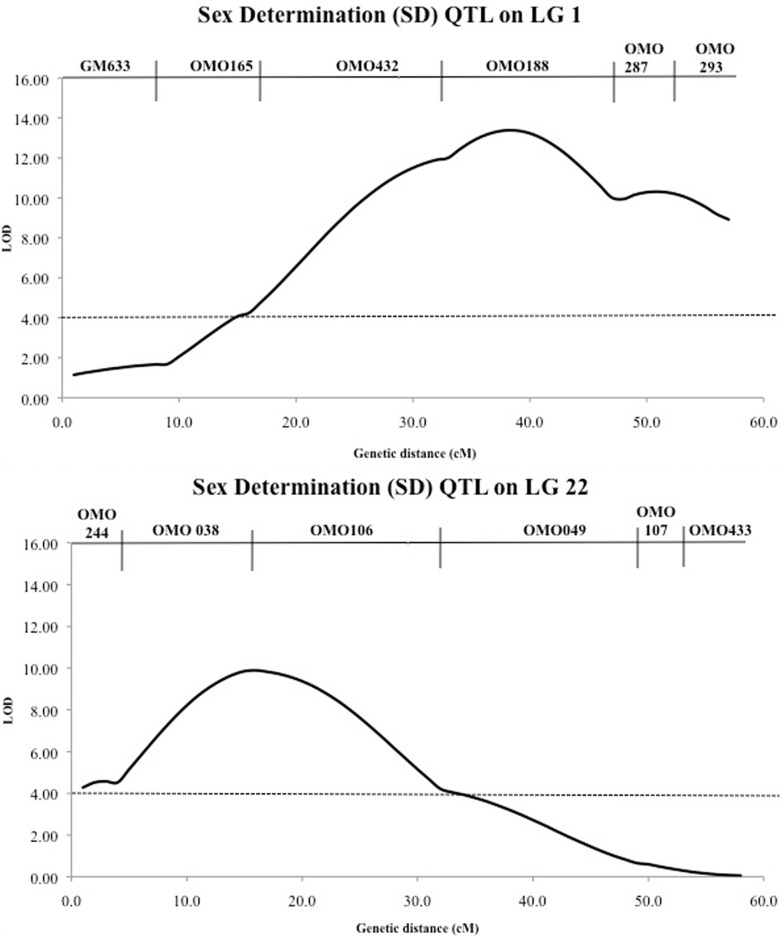
QTL for sex detected on LG 1 and LG 22 in salt-tolerant tilapia. The dotted line represents the genome wide threshold *p* < 0.05 for QTL is 4.0. The genetic distance on each linkage group is in centiMorgans (cM).

Two significant QTL for sex were detected on LG 1 and LG 22 respectively. The significant QTL detected were supported with LOD values of 13.38 and 9.89 explained 19.8% and 10.8% of phenotypic variance respectively on LG 1 and LG 22 (Fig 1, Table 2).

**Table 2:** Significant and suggestive quantitative trait loci (QTL) for growth traits and sex in all, male and female offspring of salt-tolerant tilapia.

Traits	LOD threshold	QTL ID	LOD	Linkage group (LG)	QTL interval	Peak position (cM)	PVE (%)
BW	4.0	BW	2.79*	20	8.8–15.6	11.8	3.1
m-BW	4.0	mBW-a	2.82*	2	0.0–15.0	15.0	4.7
		mBW-b	2.51*	15	0.0–21.3	4.0	6.3
		mBW-c	2.55*	17	0.0–2.2	0.0	4.1
		**mBW-d**	3.69**	18	51.4–56.4	55.4	6.2
		mBW-e	2.62*	22	11.0–27.0	17.0	5.8
f-BW	4.1	fBW-a	2.72*	1	32.6–45.9	35.6	11.6
		fBW-b	2.78*	6	0.0–8.6	4.0	7.8
		**fBW-c**	4.80***	8	3.3–16.1	4.3	12.1
TL	4.0	TL-a	2.21*	20	8.8–15.6	11.8	2.6
		TL-b	2.37*	22	12.6–31.5	22.6	3.3
m-TL	4.3	**mTL-a**	5.12****	2	0.0–15.0	15.0	8.4
		mTL-b	2.22*	17	0.0–2.2	0.0	3.5
		**mTL-c**	4.34***	18	51.4–56.4	55.4	7
		mTL-d	2.2*	22	11.0–27.0	15.0	4.1
f-TL	4.0	fTL-a	2.53*	1	32.6–45.9	35.6	8.1
		fTL-b	2.92*	6	0.0–8.6	3.0	7.9
		**fTL-c**	5.83****	8	3.3–16.1	4.3	14.3
		fTL-d	2.18*	17	6.5–8.2	6.5	5.9
BT	4.0	BT	2.34*	12	0.0–11.2	1.0	2.4
m-BT	4.1	mBT-a	3.44*	2	25.5–38.3	26.5	6.9
		mBT-b	2.81*	5	17.2–35.0	27.2	7
		mBT-c	2.77*	8	37.6–41.9	41.9	4.8
		mBT-d	3.32*	18	51.4–56.4	55.4	6.1
f-BT	4.1	fBT-a	2.61*	6	42.5–58.6	51.5	8
		**fBT-b**	3.71**	8	16.1–44.4	35.1	17.2
		fBT-c	2.63*	12	40.9–49.6	42.9	7.6
SD	4.0	**SD-a**	13.38******	1	30.7–44.8	35.7	19.8
		**SD-b**	9.89******	22	13.8–30.1	13.8	10.8

BW: body weight, TL: total length, BT: body thickness, SD: Sex determination. mBW: male body weight, mTL: male total length, mBT: male body thickness. fBW: female body weight, fTL: female total length, fBT: female body thickness. PVE: Phenotypic variance explained. LOD threshold scores are based on *p*<0.05. Significant QTL are underlined and bolded. Significance: Chromosome-wide *p<0.05, **p<0.01; Genome Wide ***p<0.05, ****p<0.01, *****p<0.005, ******p<0.001.

### Mapping QTL for growth traits

We conducted QTL analysis for growth traits in all 522 F_2_ offspring, 235 males and 164 females, respectively. In all 522 F_2_ offspring, three suggestive QTL were detected on LG 20, LG 22 and LG 12 for BW, TL and BT. The suggestive QTL explained 2.4 to 3.1% of phenotypic variance (S5 Fig and Table 2).

**Fig 2:**
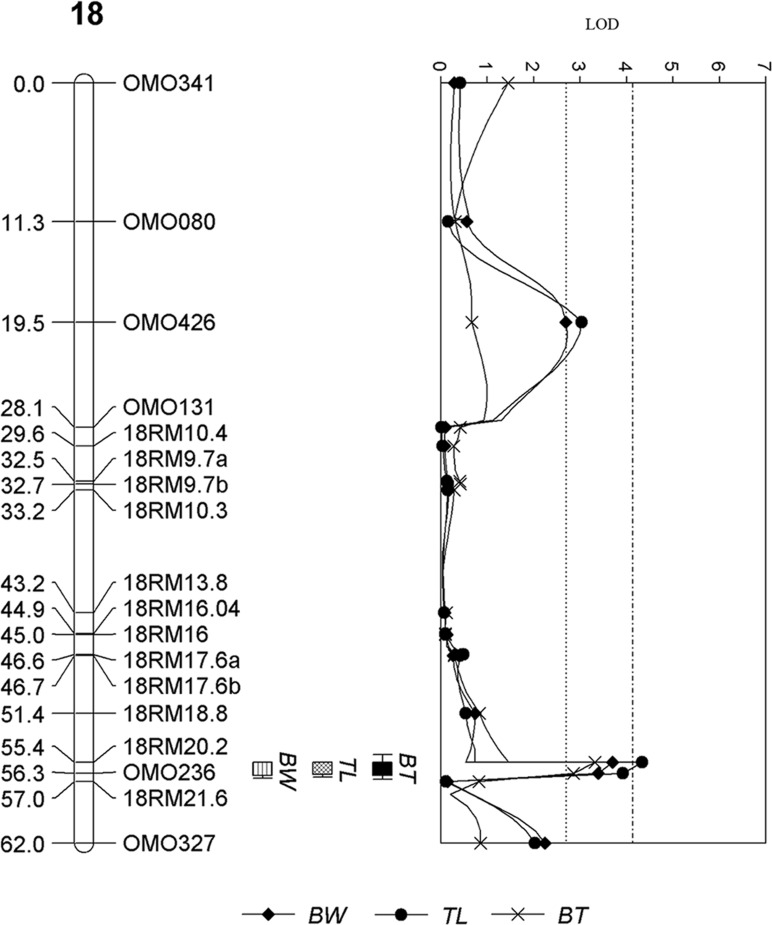
QTL for growth traits detected on LG 18 in male offspring of salt-tolerant tilapia. The number of the left is the distance in centiMorgans (cM). The labelings on the right are the names of the microsatellite DNA markers. The top of the graph is the LOD value of the QTL peaks and the dotted line of LOD value 2.8 and 4.13 represents the LOD threshold for chromosome and genome wide significance of *p*-value < 0.05, respectively. The boxes are representative of the traits associated with the detected QTL regions; BW—body weight, TL—total length and BT—body thickness.

**Fig 3:**
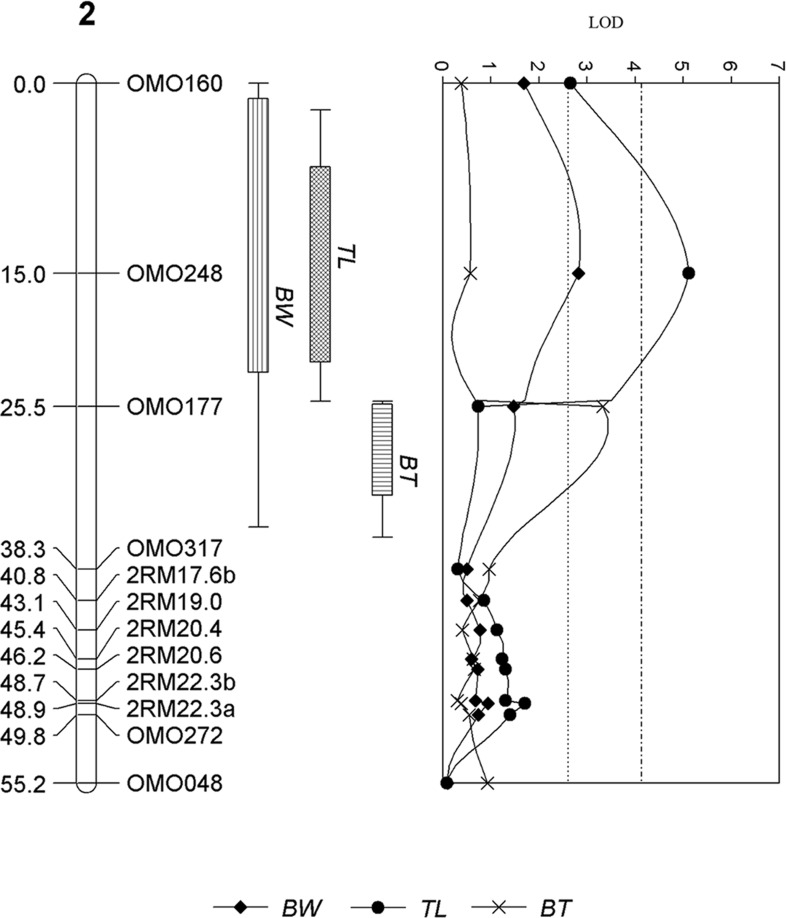
QTL for growth traits detected on LG 2 in male offspring of salt-tolerant tilapia. The number of the left is the distance in centiMorgans (cM). The labelings on the right are the names of the microsatellite DNA markers. The top of the graph is the LOD value of the QTL peaks and the dotted line of LOD value of 2.6 and 4.15 represents the LOD threshold for chromosome wide and genome wide significance of *p*-value < 0.05, respectively. The boxes are representative of the traits associated with the detected QTL regions; BW—body weight, TL—total length and BT—body thickness.

For the male offspring, a total of two significant and six suggestive QTL were detected in LG18 (Fig 2), LG 2 (Fig 3), and 2, 5, 8, 15, 17 and 22, respectively. The significant and suggestive QTL and explained 3.5% to 8.4% of phenotypic variance (Table 2). One of the two significant QTL detected on LG 18 was associated with all traits (Fig 2) while the other significant QTL detected on LG 2 was associated with TL and suggestively associated with BW (Fig 3) Another QTL detected on LG 2 located on a different location was suggestively associated with BT (Table 2). Suggestive QTL associated with BW and TL were detected in similar locations on LG 17 and LG 22. One and two suggestive QTL associated with BW and BT were detected on LG 15 and LG 5 and 8, respectively (S6 Fig and Table 2).

**Fig 4:**
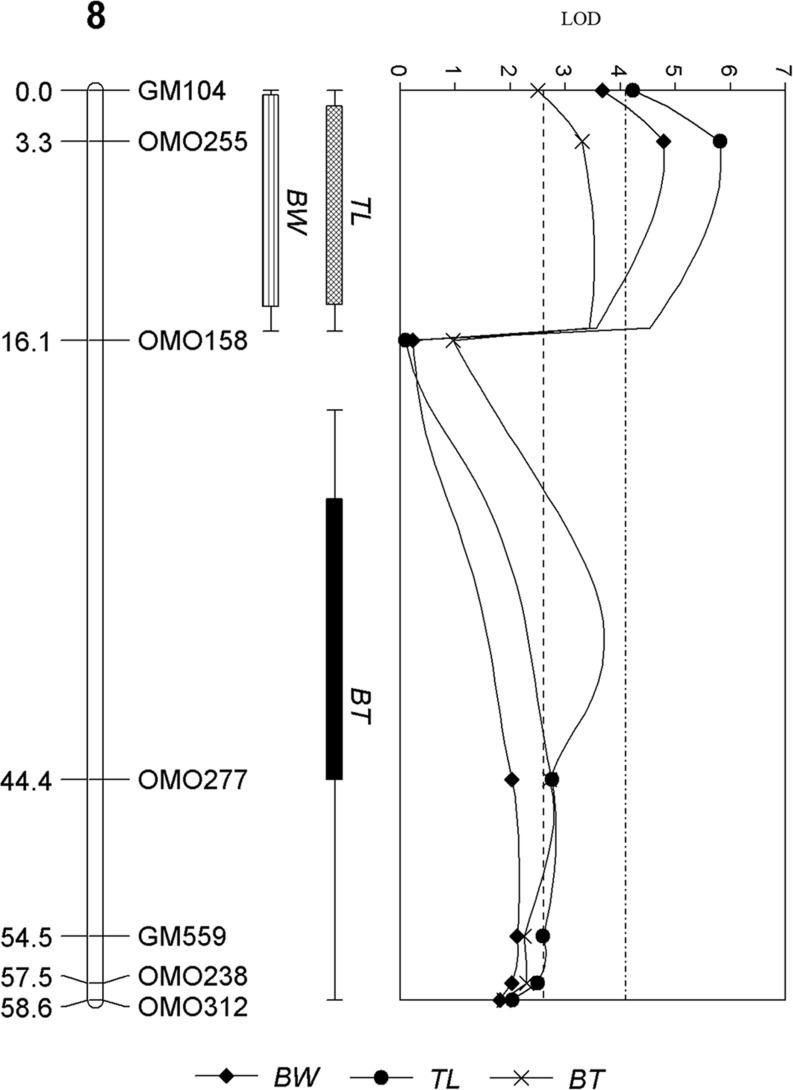
QTL for growth traits detected on LG 8 in female offspring of salt-tolerant tilapia. The number of the left is the distance in centiMorgans (cM). The labelings on the right are the names of the microsatellite DNA markers. The top of the graph is the LOD value of the QTL peaks and the dotted line of LOD value 2.56 and 4.03 represents the LOD threshold for chromosome wide and genome wide significance of *p*-value < 0.05, respectively. The boxes are representative of the traits associated with the detected QTL regions; BW—body weight, TL—total length and BT—body thickness.

For the female offspring in the population, a total of two significant and four suggestive QTL were detected in linkage groups LG 8 (Fig 4) and 1, 6, and 12 respectively (S7 Fig and Table 2). The significant and suggestive QTL explained 7.6 to 17.2% of phenotypic variance. One of the two significant QTL detected on LG 8 was associated with BW and TL, while the other significant QTL detected on a different location in LG 8 was associated with BT. Two suggestive QTL detected on LG 6 was associated with BW and TL and BT at two different locations while LG 1 and 12 were suggestively associated with BW and TL and BT respectively (S7 Fig and Table 2).

## Discussion

Growth is regulated by genetic and environmental factors [47]. Our knowledge about genetic factors is rather limited in salt-tolerant tilapia. In our study, we conducted a QTL mapping for growth traits in all 522 F_2_ offspring, only male offspring and female offspring, respectively, in an attempt to dissect the phenotypic variances of growth traits. We also mapped QTL for sex using the 399 F_2_ offspring with known sexes.

We conducted a QTL analysis for sex in an F_2_ Family containing 399 offspring with known sexes. Two significant QTL associated with sex was detected in LG1 and LG22, which explained 19.8% and 10.8% of phenotypic variance, suggesting that in our salt-tolerant tilapia family, the sex determination may be two different genomic locations. Sex determination loci were previously mapped on LG1 [27,35], LG3 [35], LG23 [36,37] in different tilapia species and in salt-tolerant tilapia, the sex determining loci have been mapped in LG22 [25]. These results suggest that sex determination in tilapia is a complicated issue and may be determined by genetic and environmental factors. In order to understand the mechanisms underlying sex determination in tilapia, more studies should be carried out.

QTL analysis for growth traits using all the 522 offspring revealed three suggestive QTL for growth traits on LG 20, LG 22 and LG 12. The effects and locations of QTL for growth traits detected in this study were rather small and somewhat different from those detected in previous studies in Nile tilapia [4,32]. This may reflect in the nature of QTL for growth traits across species and/or families within a species, but it is also possible that it was caused by different sensitivity of QTL detection in different studies. The power of QTL mapping is influenced by a number of factors such as population size, population structure, environmental effects, marker density, genotyping errors and precision of trait measurement [48,49]. Since the effect of QTL for growth traits is rather small in all offspring in this study, we conducted a separate analysis of QTL for growth in our male and female offspring, respectively. Larger effects of QTL for growth traits were detected on several linkage groups in male offspring and female offspring, respectively. This result suggests that separation of male and female offspring has the higher power in detecting QTL for growth traits than all offspring in salt-tolerant tilapia. We found that the identified QTL for growth traits were located in different linkage groups in male offspring and female offspring, suggesting that in our mapping family, males and females use a different set of genetic ‘switches’ to control growth besides sex. Further fine mapping of these QTL for growth in males and females may shed more lights on the growth variance in the salt-tolerant tilapia.

In male offspring, significant QTL were detected on LG 2 and 18. These LGs correspond chromosome groups 4 and 3 of the stickleback [26] and were reported to be associated with gain and lost of most of the skeletal related traits in the stickleback [50]. In addition, the transferrin gene, an iron-binding glycoprotein, previously mapped onto LG 18 [51], has been known to play an important role in the osmoregulation and salt tolerance [52]. Therefore, it is possible that the genes underlying the QTL regions of LG 2 and LG 18 may have a pleiotropic effect on multiple growth traits in male salt-tolerant tilapia or that these genes are tightly linked to genes that confer such phenotypes in the population. Further experiments on the effect of epistasis on the phenotypes, fine mapping and positional cloning will be required to understand the functions of genes responsible for growth.

QTL for body weight detected on LG 22 were significantly and suggestively associated with sex and TL in all offspring, respectively, as well as with both BW and TL in the male offspring. Additionally, QTL detected on LG 1 was significantly and suggestively associated with sex and BW and TL in female offspring. This may be indicative of the interplay between genes that is underlying the sexual dimorphism of growth in tilapia as LG 1 and LG 22 have been reported to be associated with sex determination [25,27,35]. Suggestive QTL detected on LG 15 in the male offspring have evoked a particular interest, as estrogen receptor (ER), found to be gender specific and expressed higher in males was reported to map onto LG 15 [26]. Altogether, these LGs may be suggestive of the pleiotropic effects that may be affecting the growth of fishes in a male-specific and family-specific manner. Most of the suggestive QTL that have been detected in our population have not been studied in detail. Two QTL of genome-wide and chromosome-wide significance for growth traits in LG 8 and LG17 were detected in both male and female offspring and account for 4.8% to 17.2% and 3.5% to 5.9% explained variance in male and female offspring. Given the polygenic nature of growth traits [49], the genes residing in these QTL regions could play a minor yet complex role in conferring the varying phenotypes of growth in the population.

In summary, we conducted a QTL mapping for growth traits and sex in an F_2_ family of salt-tolerant hybrid tilapia. Utilizing a single family for our QTL detection may allow us to detect family-specific and rare QTL [53] since phenotypic divergence is present in the genetic background of parental strains, being previously selected for salinity tolerance and quick growth [4,25]. Additionally, sexual dimorphism of growth traits is evident in our mapping population as the QTL for sex were located on LG 1 and 22. The QTL for growth traits in males and females were located in different LGs, suggesting that in salt-tolerant tilapia, different set of gene ‘switches’ control the growth in males and females. Since all male production is preferred in production, the QTL identified in our study provides a good starting point in the marriage of breeding of monosex cultures with good growth performance. Consequently, efforts will be made to conduct QTL for growth and sex in more families and to validate the identified QTL in our study. The approach of using multi family mapping may allow us to evaluate and cross validate the identified QTL due to the different genetic backgrounds and increase the resolution of the QTL positions and the accuracy of the QTL effects [54]. Our future efforts will be focused on utilizing NGS platforms such as genotype-by-sequencing (GBS) [55] to increase the efficiency and power of QTL mapping of growth and sex through an increment in the density of markers per linkage group. Our study sets the foundation for further fine mapping QTL, positional cloning and identification of possible candidate genes for growth, sex determination and MAS in salt-tolerant tilapia.

## Supporting Information

S1 Fig A genetic linkage map of salt-tolerant tilapia constructed using all offspring. There are 22 linkage groups named with LG1-22. The number of the left is the distance in centiMorgans (cM). The labeling on the right are the names of the microsatellite DNA markers (see S1 Table).
(TIF)

S2 Fig A genetic linkage map of salt-tolerant tilapia constructed using male offspring. There are 22 linkage groups named with LG1-22. The number of the left is the distance in centiMorgans (cM). The labeling on the right are the names of the microsatellite DNA markers (see S1 Table and S3 Table).
(TIF)

S3 Fig Genetic linkage map of salt-tolerant tilapia constructed using female offspring. There are 22 linkage groups named with LG1-22. The number of the left is the distance in centiMorgans (cM). The labeling on the right are the names of the microsatellite DNA markers (see S1 Table).
(TIF)

S4 Fig Genetic linkage map of salt-tolerant tilapia constructed using male and female offspring. There are 22 linkage groups named with LG1-22. The number of the left is the distance in centiMorgans (cM). The labeling on the right are the names of the microsatellite DNA markers (see S1 Table).
(TIF)

S5 Fig QTL profile for growth traits and sex determination in all offspring. Body weight–BW, Total length–TL, Sex determination–SD. Dotted line represents threshold of genome wide significance of *p*<0.05.
(TIF)

S6 Fig QTL profile for growth traits in male offspring. Body weight–BW, Total length–TL, Body thickness–BT. Dotted line represents threshold of genome wide significance of *p*<0.05.
(TIF)

S7 Fig QTL profile for growth traits in female offspring.Body weight–BW, Total length–TL, Standard length–SL, Body thickness–BT. Dotted line represents threshold of genome wide significance of *p*<0.05.
(TIF)

S1 Table Selected markers for reconstruction of a linkage map for QTL mapping in salt-tolerant tilapia. (DOCX)

S2 Table Primers of additional 18 microsatellite markers for fine mapping QTL for growth traits in male offspring of salt-tolerant tilapia. (DOCX)

S3 Table Phenotypic correlations between recorded growth traits of salt-tolerant tilapia at180 dph. (DOCX)

S4 Table Summary of statistics for the genetic linkage maps for QTL mapping in salt tolerant tilapia; total length, number of markers and average marker distance. (DOCX)

S5 Table Recombination ratios and shared markers between female and male offspring maps of salt tolerant tilapia. (DOCX)
