# Supplementary figures and images for: Mapping QTL for Sex and Growth Traits in Salt-Tolerant Tilapia (Oreochromis spp. X O. mossambicus)
Source: PLoS One. 2016 Nov 21;11(11):e0166723. doi: 10.1371/journal.pone.0166723 (PMC5117716; doi:10.1371/journal.pone.0166723)

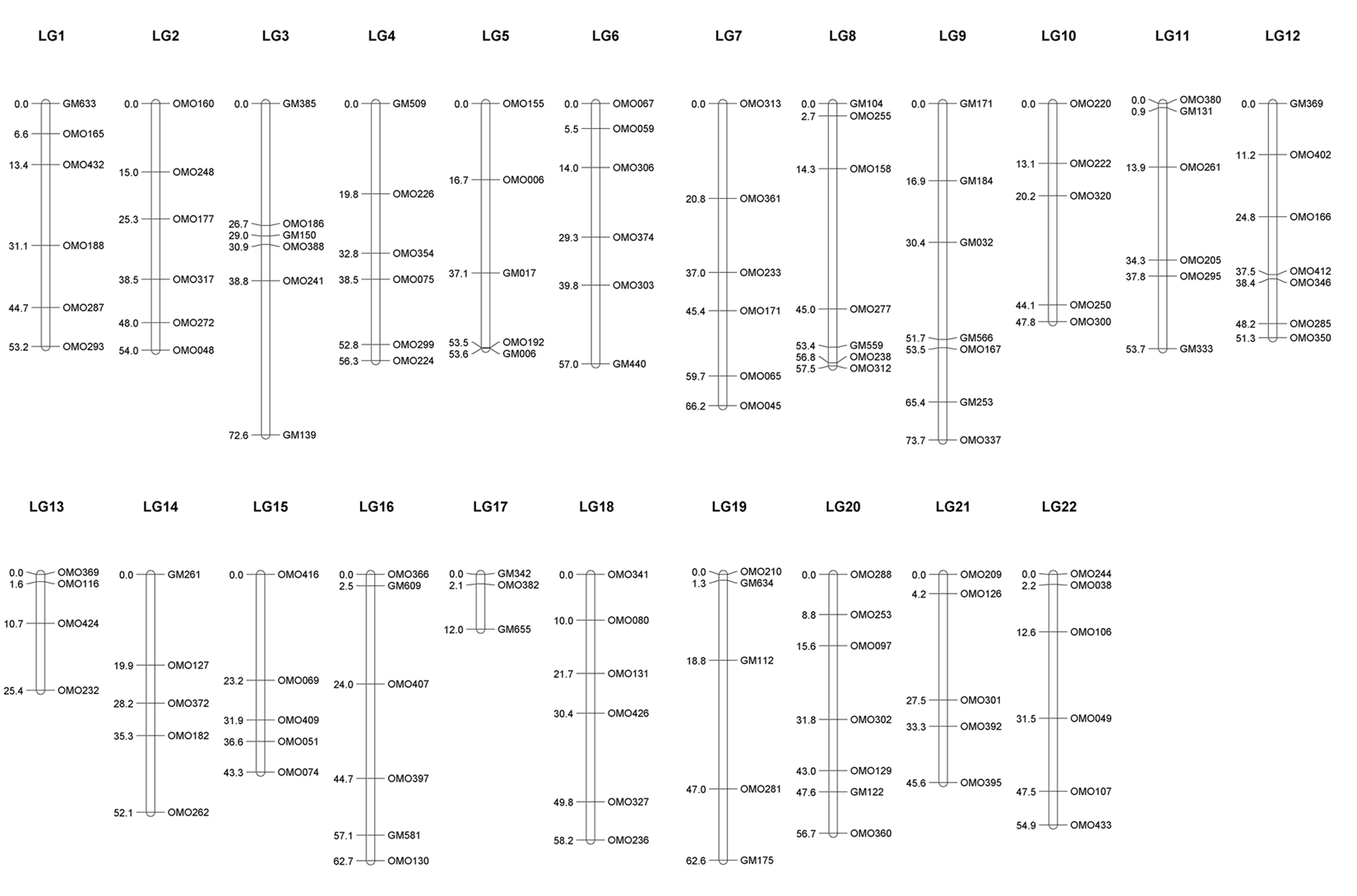

Supplement: S1 Fig — There are 22 linkage groups named with LG1-22. The number of the left is the distance in centiMorgans (cM). The labeling on the right are the names of the microsatellite DNA markers (see S1 Table). (TIF) [file pone.0166723.s001.tif]

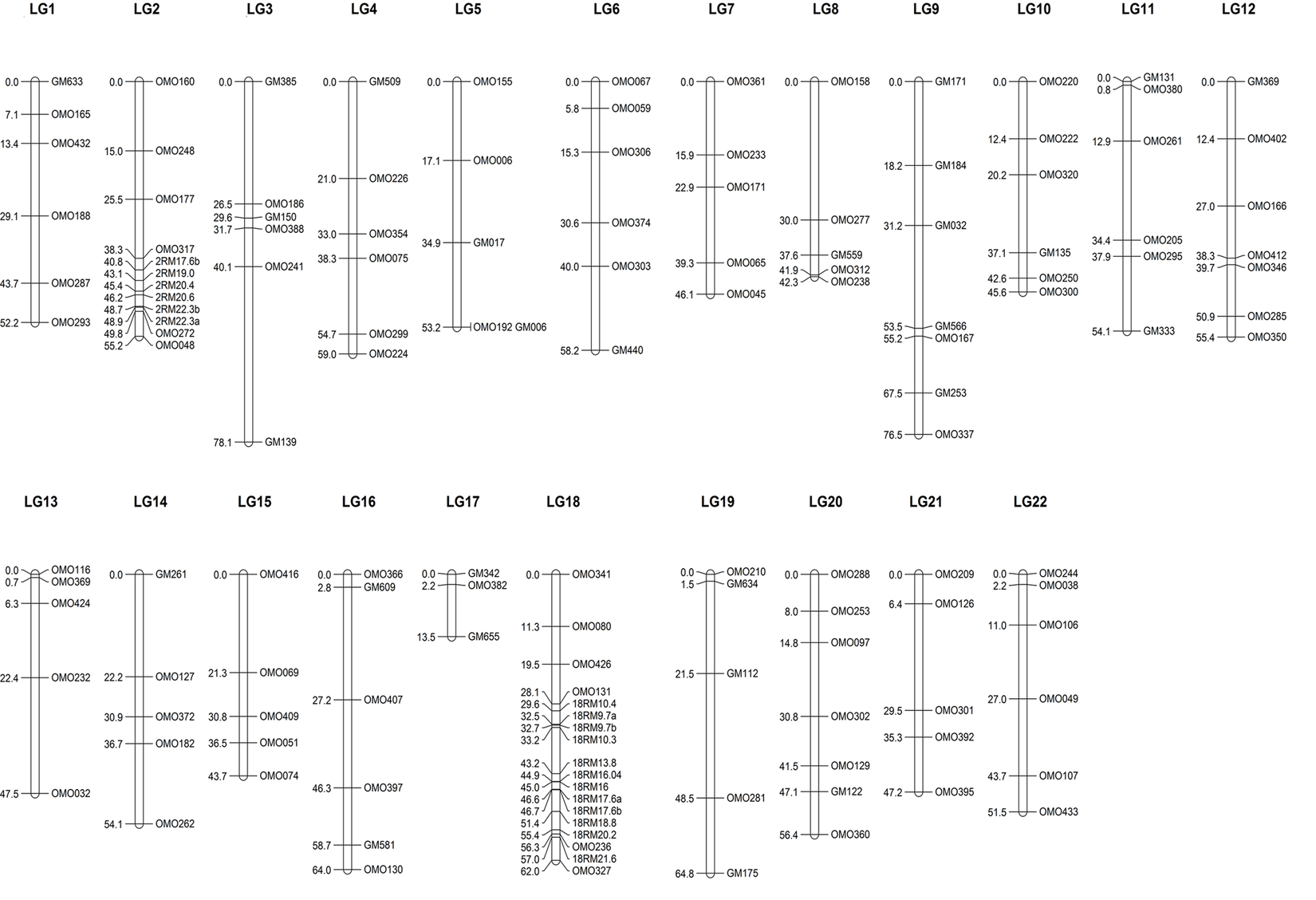

Supplement: S2 Fig — There are 22 linkage groups named with LG1-22. The number of the left is the distance in centiMorgans (cM). The labeling on the right are the names of the microsatellite DNA markers (see S1 Table and S3 Table). (TIF) [file pone.0166723.s002.tif]

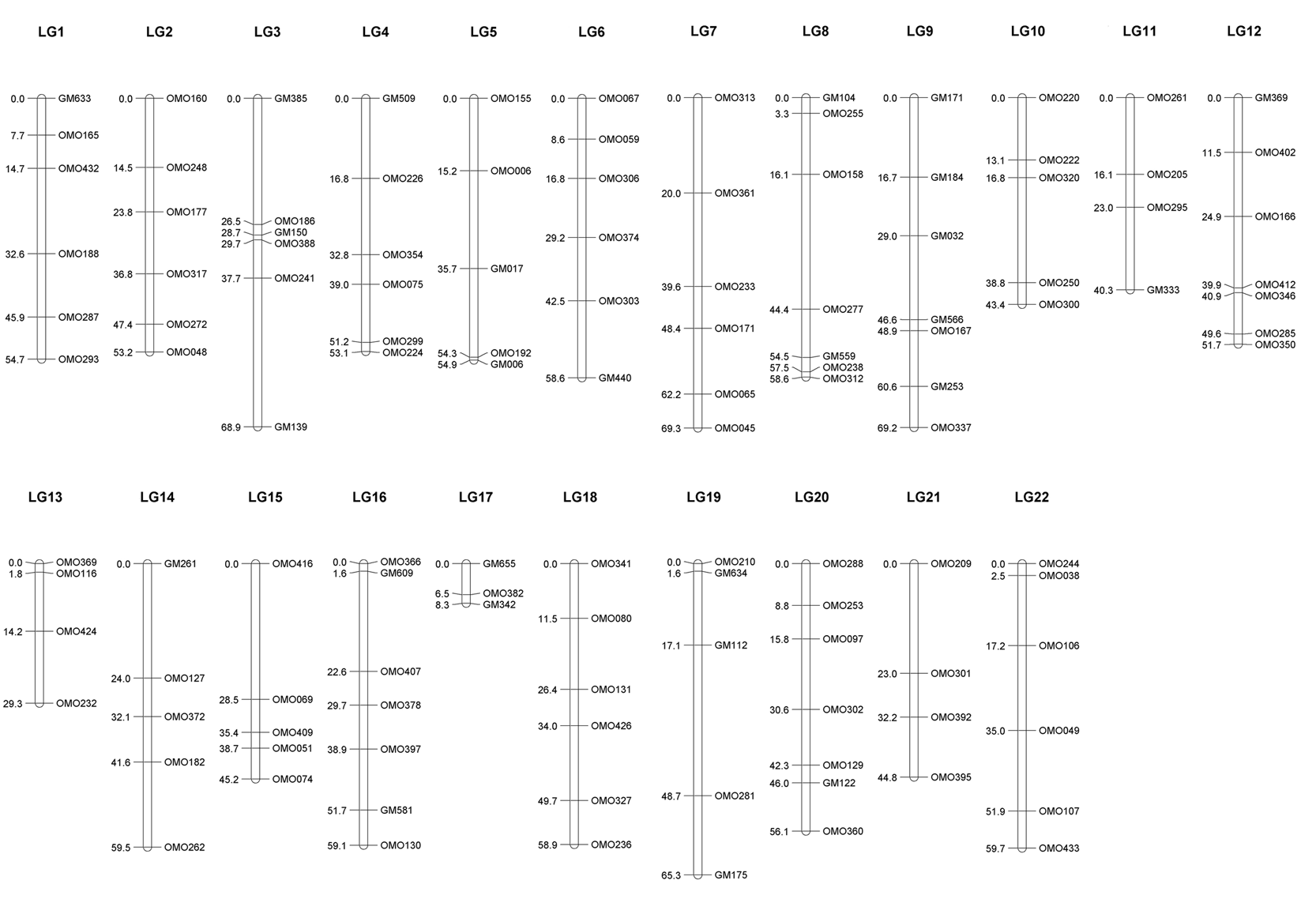

Supplement: S3 Fig — There are 22 linkage groups named with LG1-22. The number of the left is the distance in centiMorgans (cM). The labeling on the right are the names of the microsatellite DNA markers (see S1 Table). (TIF) [file pone.0166723.s003.tif]

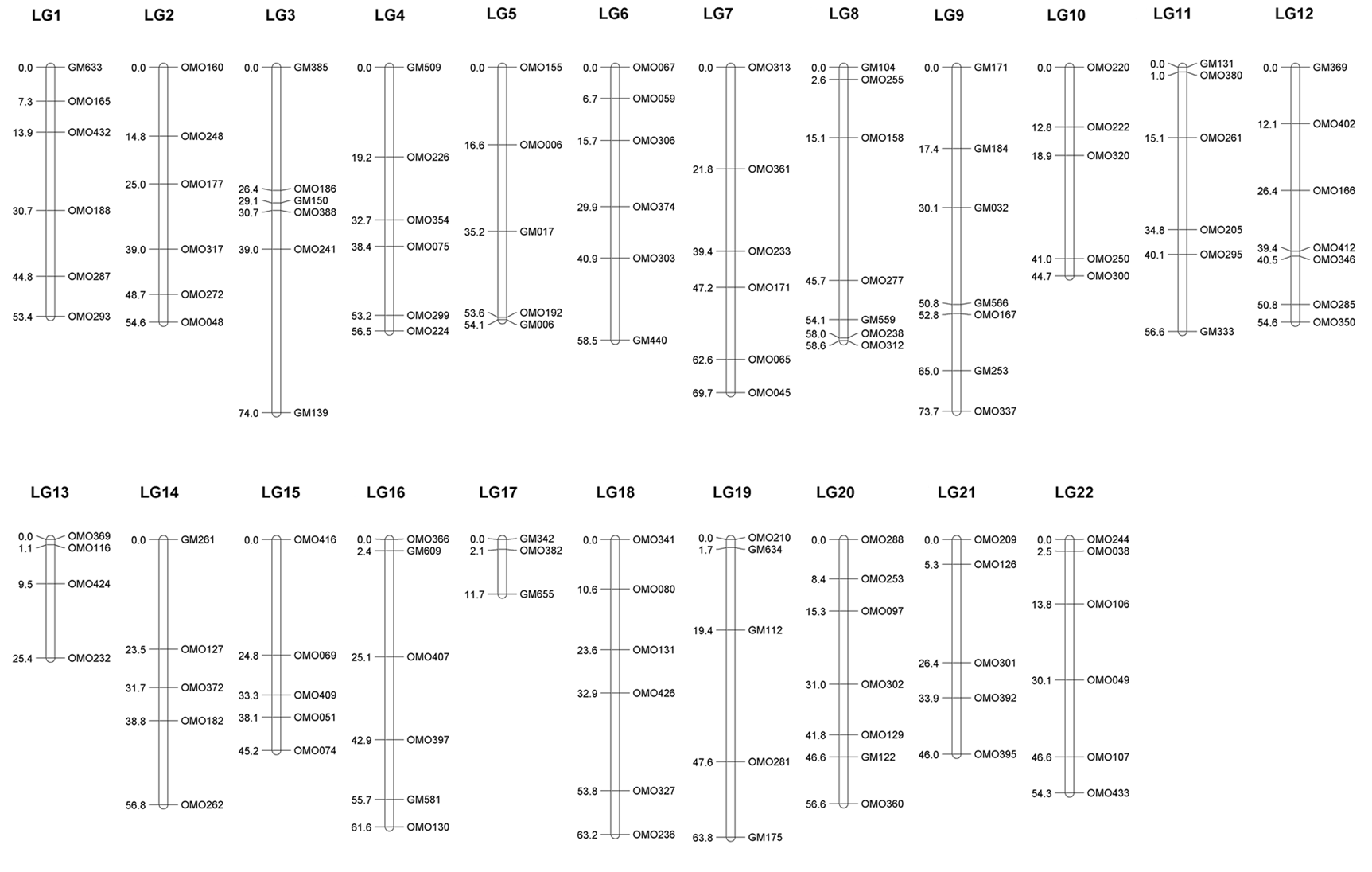

Supplement: S4 Fig — There are 22 linkage groups named with LG1-22. The number of the left is the distance in centiMorgans (cM). The labeling on the right are the names of the microsatellite DNA markers (see S1 Table). (TIF) [file pone.0166723.s004.tif]

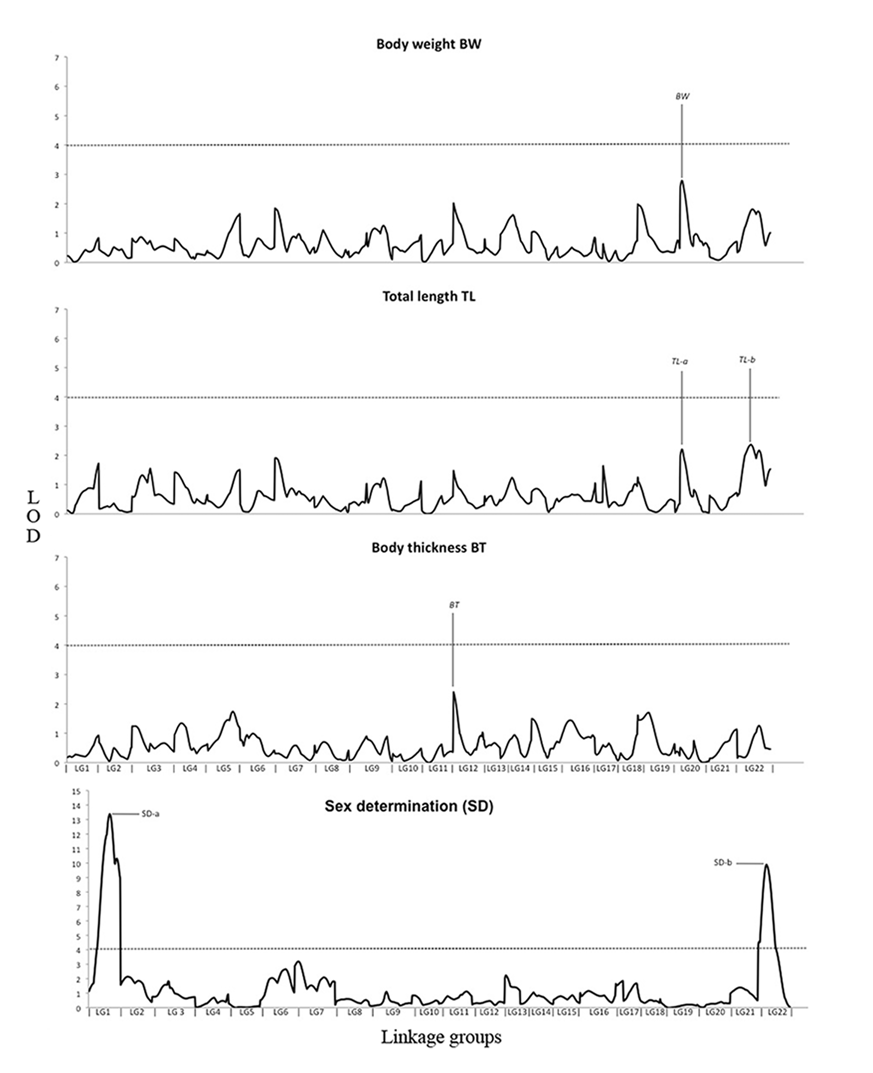

Supplement: S5 Fig — Body weight–BW, Total length–TL, Sex determination–SD. Dotted line represents threshold of genome wide significance of p<0.05. (TIF) [file pone.0166723.s005.tif]

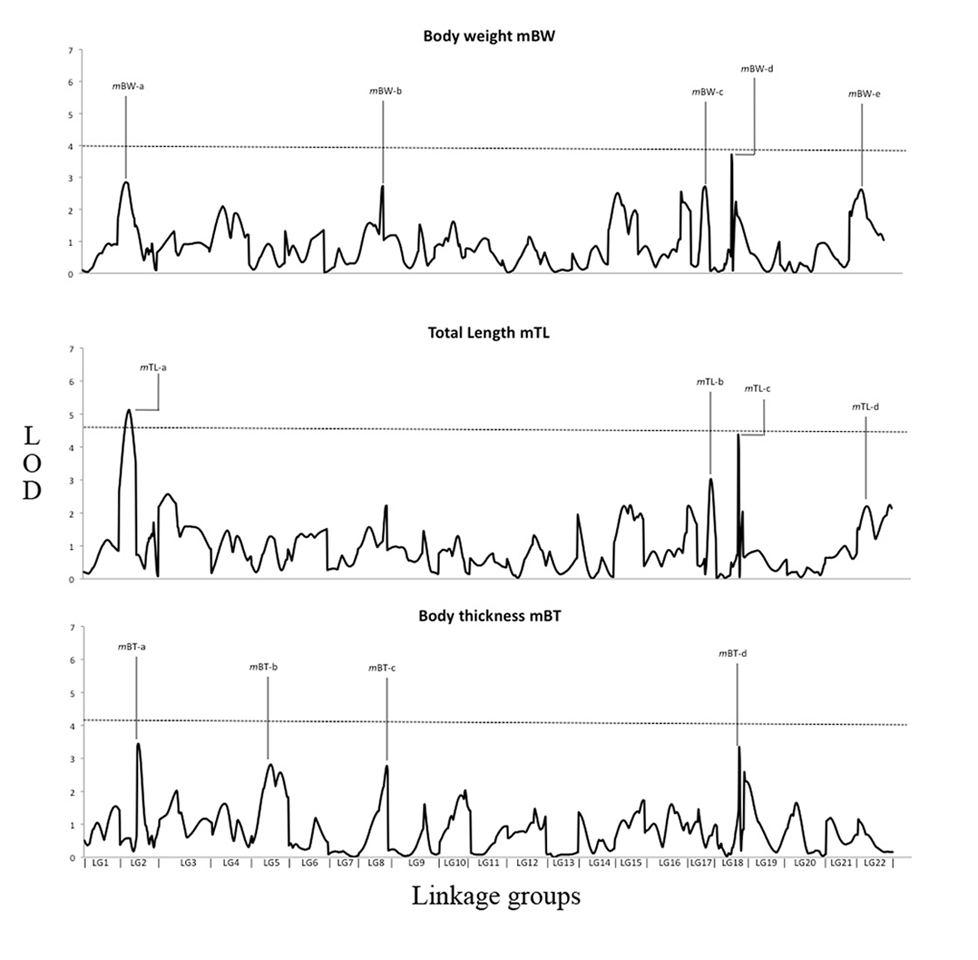

Supplement: S6 Fig — Dotted line represents threshold of genome wide significance of p<0.05. (TIF) [file pone.0166723.s006.tif]

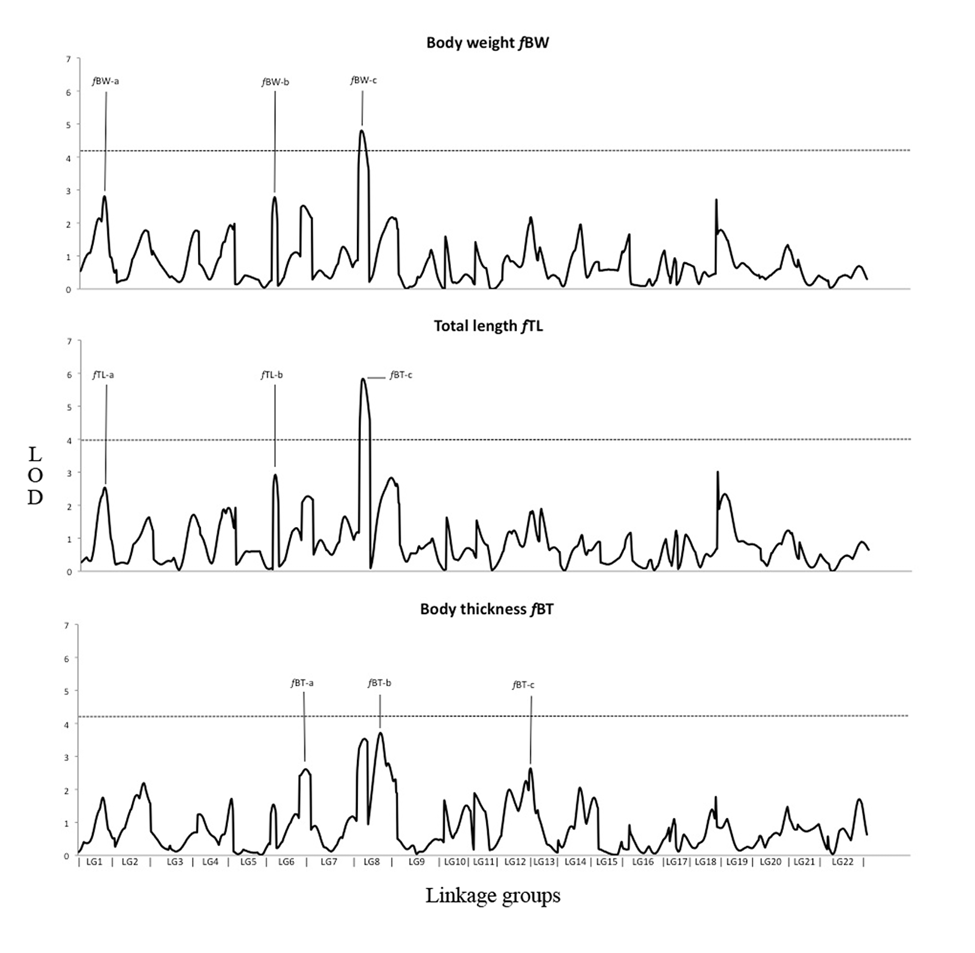

Supplement: S7 Fig — Dotted line represents threshold of genome wide significance of p<0.05. (TIF) [file pone.0166723.s007.tif]
