# Supplementary material for: Mapping QTL for Sex and Growth Traits in Salt-Tolerant Tilapia (Oreochromis spp. X O. mossambicus)
Source: PLoS One. 2016 Nov 21;11(11):e0166723. doi: 10.1371/journal.pone.0166723 (PMC5117716; doi:10.1371/journal.pone.0166723)
Supplement: S1 Table — (DOCX) [file pone.0166723.s008.docx]

| S1 Table : Selected markers for reconstruction of a linkage map in salt-tolerant tilapia | | | |  |  |  |
| --- | --- | --- | --- | --- | --- | --- |
| **Linkage group** | **Marker name** | **Primer sequence (forward)** | **Primer sequence (reverse)** | **Motif** | **Product length (bp)** | **Annealing temperature** |
|  |  |  |  |  |  | **(^o^C)** |
| 1 | GM633 | GACCCAGGACTCATGTGCTT | GTGTCCCAAGAAAACCAGGA | (CA)27 | 178 | 55 |
| 1 | OMO165 | AAAGCGAGGATCAGCCAGAGTTAC | TGACGGGGTGAACAAAATAAAAGA | (CA)10 | 287 | 60 |
| 1 | OMO188 | ACATATTCGCCTTGTTCTCACCAG | GAAGTGTGCGTTCAAAGTATGATT | (CA)16 | 163 | 55 |
| 1 | OMO287 | AACCTGGTTAGTCTCTGGCTGCTC | ATGGCATGGTTTGCAACATAATCC | (GT)17 | 280 | 60 |
| 1 | OMO293 | CTATCTGCCCCGGTTTCTTTGT | ATGCCGCATTTTTAGTTTCCATA | (GA)23 | 435 | 60 |
| 1 | OMO432 | AGTAGTTTTTGTTCATTAGAGACG | TAGTCACACTGATAATTTTTCTTG | (ATAG)15 | 224 | 50 |
| 2 | OMO048 | TCCCCACACCTCCTGCTCCTC | AGCCCAAGCCAGTCCCATAGC | (CA)18 | 336 | 60 |
| 2 | OMO160 | AGGATTTCCTGAAAGTGTTTTT | ACTCTACGTGACCTCTGACAATAG | (GT)10 | 210 | 55 |
| 2 | OMO177 | AGTGATGACCGGCCAGAAAGAGA | CAGGGATGGATAAACGTGACAATG | (CA)11 | 345 | 55 |
| 2 | OMO248 | AAAGACACAAAGAGAAACTAATCA | GGATGAATATTTAAAATCAGTCAG | (TCA)9 | 381 | 55 |
| 2 | OMO272 | CAGGGCTCATGGTAACAGTTCATA | TAGTACGAAGCGCTAGCAGTCTCT | (GT)23 | 454 | 60 |
| 2 | OMO317 | ACATCTACTGGTATCTGGGTTCAA | GTTTAATTCTGGGAGGGCACAC | (CA)32 | 443 | 60 |
| 3 | GM139 | TTTGAGTAACCACCCTAACAC | GTGGGATCTACCAAGAAGAG | (GT)17 | 255 | 55 |
| 3 | GM150 | AGGTGATTGGCTTAGATGAT | GTCTCAGTTTGTTTGGCTTA | (CA)30 | 177 | 55 |
| 3 | GM385 | GTAAAGCAAAACTCAGAGGGT | AATGATCCCGGCTGTT | (CA)10 | 282 | 55 |
| 3 | OMO186 | CAAATGAGCGAAGAAAACAATAAA | TCCCTCTCCCATCTGAGTCAAGTA | (GT)10 | 333 | 55 |
| 3 | OMO241 | AGAGTACGACCCTGCACACTTCAC | TGATCAACAATCTGGCTTATTCTG | (CA)23 | 219 | 50 |
| 3 | OMO274 | TCAGCTGGATCTGCTCAGTGTGAC | ACAGAACGCAGTGCTTTCATTTTG | (CA)9 | 401 | 60 |
| 3 | OMO388 | GGGATTTGTTTTCCTCCTTAGCA | CAGCCATTGTTGTGTATTTGTGAC | (GT)7 | 143 | 55 |
| 4 | GM509 | AGCCCTGCACAGTTTAGCAA | CAACAGCAAAGCCAGGAGAG | (CA)23 | 181 | 55 |
| 4 | OMO075 | CGGCTCGGCCTCCTCATCT | TAGGCACAAAGTGGGTGGAAGGT | (CA)13 | 464 | 55 |
| 4 | OMO092 | GCACGCTGAAACTTACACAGTGA | GAGGCTTAGTTGGGCTGATTGATT | (CT)12 | 185 | 60 |
| 4 | OMO224 | TTCCCGGAGTACAGAATGTTAGAC | GAGGCTGCATTAGCTAGTGGATAC | (GT)13 | 260 | 55 |
| 4 | OMO226 | ACGAGCCAACCTGCGAAGAGAT | GCTGCTCAAGCCAAAAATGTCAAA | (GT)22 | 451 | 55 |
| 4 | OMO299 | GTTGGACAACATCGCCTACATCAT | CACGCAATTAACGTCCCACTGAA | (GT)10 | 365 | 60 |
| 4 | OMO354 | AAGTATAAACCCCCGTGCGTGAG | ACAGGGGAGGTGGAGGAGGAA | (CA)14 | 342 | 60 |
| 5 | GM006 | AGCCAACAACTATTCTGCTGT | TCGGGTTGTGCTCCTTAG | (CA)16 | 93 | 55 |
| 5 | GM017 | GATACCTGTCCATACCTCCTC | CCCTCTGTTTCCATCTCA | (CA)26 | 160 | 55 |
| 5 | OMO006 | TTGGCCTTTTCTCTGGGGTTATT | TGGCCCGTTAAACTCTGAAAACAT | (CA)10 | 435 | 55 |
| 5 | OMO024 | AGTTTCTGCTGCTATTCATTAC | GGCTCAAGATACCCATACAA | (CA)27 | 403 | 60 |
| 5 | OMO155 | AGATCTCCCCGAAACCCTGATTAT | CAAACGCTACGCTGTGCTGCTA | (CA)12 | 206 | 60 |
| 5 | OMO192 | TTTCCGCACATTAATCTCAATACA | ATCTTTCATCTGCTCACCGTCTCT | (AT)9 | 399 | 60 |
| 6 | GM440 | CTGCACTTTTACTGAGGG | TGGGAGATTAACAGAATAAC | (CA)21 | 264 | 55 |
| 6 | OMO059 | TCCACGATGGGATGAACCTTGAG | TGTTGCCGTGTGACTGTTGCTCT | (CA)10 | 179 | 60 |
| 6 | OMO067 | GTTTCTCGTCCCAAAGTCTCACAG | TCCAGATTCGCTTTAGTTTGTTCA | (GT)8 | 179 | 60 |
| 6 | OMO265 | CAACCACTGCAACCACGTAAAGAG | CCCAGGCCAGCTGACAGAAA | (GT)8 | 110 | 60 |
| 6 | OMO303 | CTCACCCGAGCAGGCAGGAG | GCTGATGTTCTGGGGGATAATGG | (CAG)7(CAA)10 | 181 | 55 |
| 6 | OMO306 | AATGCGAAGGCGAGTGGAGTG | AGCAGGTGATTAGGAGAGGCTGAT | (GT)10 | 182 | 60 |
| 6 | OMO374 | GTAAGCGCAATGATATGAATGAAT | TTGCCAAGATATTAGAACCAGATG | (CA)12 | 409 | 60 |
| 7 | OMO045 | ATCTCCCAAAGACATGCAAAAGAA | ATAAGCCTCAGTACCCCCACCACT | (AT)8 | 417 | 60 |
| 7 | OMO065 | AACGGTTTAACAGTCACGCACATC | TGCTTACTCGCCATTGACATTCTT | (GT)9 | 240 | 60 |
| 7 | OMO171 | AGCCTCGGGAAGAGTGAAGTCAAC | TTTTTGCTAACCCATCTCCTGTGA | (CT)9 | 153 | 55 |
| 7 | OMO233 | TGTTCAGACTGCAGCTTCATTAGA | CAGCCTGCTGGTCTGGAAATA | (GT)19 | 336 | 60 |
| 7 | OMO313 | GGGGGTGCAATAAAGGCTGAG | CTGCAAGGCACAAACTATCAATAA | (TA)8 | 445 | 60 |
| 7 | OMO361 | TGACAGCGAGCCAGAATGGAAGTA | AAAAGTGAAAGGGGCACAGTGAGG | (CTT)17 | 450 | 55 |
| 8 | GM104 | ACTAACCCCTGCTCTGTGCTT | GAACCCAGCGATGTCCC | (GT)15 | 273 | 55 |
| 8 | GM559 | ATTTTCCACCGAGCTCACCT | GCACCATTTTTAACCAGTGC | (GT)24 | 134 | 55 |
| 8 | OMO158 | CTTGCAGACGGTTCATTTCACGAG | TCCGGTTAATCGCTCTCACTCTCA | (GT)18 | 425 | 55 |
| 8 | OMO238 | ATATTACGTCCAAACATCCAGAGC | GAGCCAAAGGCAGAAGTAAACAGT | (CT)11 | 208 | 55 |
| 8 | OMO255 | GAGTTTTGAGCCAGCATGATTTCA | AAAAGGGGACAGGTCACTGGATTC | (AT)7 | 356 | 60 |
| 8 | OMO277 | TGAAGCAGTGTCAGCAGAGG | TTTTTCCTTTTAGGTGTTTGAG | (GAA)9 | 133 | 55 |
| 8 | OMO312 | ATAGTTTGGCAGGTCATTTTCAGA | GGGGTAGTTTTGTTGTGCTTTTT | (GT)30 | 345 | 55 |
| 9 | GM032 | TTTCGACAAGAAGGCGTTGA | CGCAGAGATTTTGGCTCATT | (CA)15 | 244 | 55 |
| 9 | GM171 | GAGTTAGGGCAGAGGTTATGT | GCCATCACCTCTTGCTT | (CA)22 | 280 | 55 |
| 9 | GM184 | GGAGAACAGGGCTTATTG | CACTTCCACCTTTACTTTGA | (CA)22 | 209 | 55 |
| 9 | GM253 | CAGAGTGATGCGTCTTCC | GCAAATACAGCACCATTCTA | (CA)24 | 218 | 55 |
| 9 | GM566 | TCTTCCGTTTGACTCACCAA | CAGACACGCAGAGATGTGGA | (CA)38 | 178 | 55 |
| 9 | OMO167 | TCACGGACACTGAACAAAGAGGA | TGGCCATGGAGATTTAAAGAACAC | (CA)23 | 393 | 60 |
| 9 | OMO337 | TAGGAGAGGCATAGGTTGTCAAAT | CAAGAGTCTAGGAGGGAATCAAAA | (GTTT)7 | 151 | 55 |
| 10 | GM135 | ATGGAAGGTCGCCCTAATAAG | GGTCCAGACACGGTCGTC | (CA)27 | 262 | 55 |
| 10 | OMO220 | ACCGCCCCTTCCTCTACCTGA | TGTGTTTGTGTATCCGCCGAGAT | (CA)13 | 408 | 55 |
| 10 | OMO222 | AAGCGATAACGGAGATTCTTCTGA | TAAAGGGTGCCGGTAAATGTAAAT | (GT)9 | 178 | 55 |
| 10 | OMO250 | TCCGGCTGCTCCAGTGTGC | GGCTGACGGAAAAATGGCTCAT | (GT)15 | 428 | 60 |
| 10 | OMO300 | TTTGCACCCTGTACTCTCACTGTA | ATATCGGGTAACATTCGCTCCACT | (CA)17 | 399 | 60 |
| 10 | OMO320 | TTTTTGGCAAGCTGGTGTAGGA | TGGACAAAGTTATGGGACCTCTGA | (GTA)8 | 367 | 60 |
| 11 | GM131 | AAAATGGCATGAAAGAACTAA | GCCATTGATTTGTTGACACT | (CA)21 | 227 | 55 |
| 11 | GM333 | CTGCGTGAATTAGCAAAATA | AAAATAAGCAAAGTGCCATA | (CA)23 | 176 | 55 |
| 11 | OMO205 | GAGAGGGAGGGATGCTTGTT | TTTGAAATTCCCACATTACATCTT | (GT)11 | 269 | 55 |
| 11 | OMO261 | GCACCGATTAGTTTGCTTCATT | CAACTGCTTTGGGGTTTATTTTAC | (CT)24 | 107 | 60 |
| 11 | OMO295 | CAAAGCGATTGATCGGAGTCTGG | CTCCCTCTCAAAACCCGATGTTAT | (ATG)7(ATT)9 | 434 | 60 |
| 11 | OMO380 | CTCGTCCTGCGTTGCCTTTTT | CAACAGCCTCCTGACCTTTTCCTA | (TTA)7 | 453 | 60 |
| 12 | GM284 | CGCAGCTTTGATTCATACCCT | AGCCGGTTTCCAGTGAGAA | (CA)16 | 177 | 55 |
| 12 | GM369 | TTTAACGAGTCTGGCATCTGA | TGCATTCATCCCATCACC | (CA)25 | 208 | 55 |
| 12 | OMO166 | CACCTCATCGGGAGACAGACAAG | TGATGGCCACTAACTCGTGAAAAA | (CA)15 | 307 | 60 |
| 12 | OMO285 | GACAATCACTCCTCCAGCCTTACC | CAGACGCTCAACTCACAGGAAAAA | (CA)10 | 285 | 60 |
| 12 | OMO346 | GACGTGAGCGGAGCTTTGTTTG | CTTCTGATGCGGTGAGGTGTTTTT | (GT)13 | 451 | 60 |
| 12 | OMO350 | GCCAACTTTCATGTACATACCAAT | ATATCATTCCCAAGTGCTCTCATT | (AT)8(GT)6 | 327 | 60 |
| 12 | OMO402 | GGGTGGAGGTTTTAACAGTTGATG | TTGGCTCATTTTGTTACCGTGATG | (GT)9 | 369 | 60 |
| 12 | OMO412 | TTTTTGTGGATTTCGGTGAGG | GCAGCGTACCAGAGTTCAGG | (GT)8 | 278 | 55 |
| 13 | OMO032 | GGTAAGCGGTGCAAAGTTCA | GTTGGCTAACCTCTGTTCACTCC | (GAAAA)10 | 313 | 55 |
| 13 | OMO116 | TGAAAATCAGCAGCACTTCGTT | TTGGGGTATTTTATGTTGGCATCT | (CA)9 | 365 | 60 |
| 13 | OMO184 | GGGGGCATGATTTACTTCTGAGGT | AAGGCTGGAGCACAGGGACTTT | (CA)12 | 182 | 60 |
| 13 | OMO232 | CATGAGCAAACAACAAACAGTGAA | GAGGCAAAGGAAGCAAGGTGAAG | (GAAA)9 | 470 | 60 |
| 13 | OMO369 | GAGTCCCAGGACCCTCTCAACC | TCATAGTGCAGCCCAATAGTGTCA | (CA)9 | 466 | 60 |
| 13 | OMO391 | AGACATCTGTACGCTCTTTACGAA | AGTGCTAGAGGGAAGGGGCTGTA | (GAT)9 | 290 | 60 |
| 13 | OMO424 | GTCGGCTGATGGGTGTAGTGGAG | AAAGGCCGTGGGGAGGTGAG | (CA)13 | 146 | 60 |
| 14 | GM261 | GCTGTAGTTACCACGAGGTGA | GTTTGGCAGCGTGTCAG | (GT)33 | 131 | 55 |
| 14 | GM276 | TCAAGTTGCGTCTCTGTCACC | CGCAGGAGGCTTTACCACA | (GT)16 | 247 | 55 |
| 14 | OMO127 | ATTTGGGTCATGAATTGGGTTTG | TGGACTGTTGCCCTTTAAGTGTTT | (GT)15 | 359 | 60 |
| 14 | OMO182 | CTCTCACTTTCACTGGGAAGGATA | AGTTCTTAGGATGCGGTCTGTCTG | (CT)20 | 190 | 55 |
| 14 | OMO262 | GCGTAGCAGCGGTGGTGTATTTA | CACCCCTGCAGTCATCCTCTACTT | (CA)10 | 161 | 60 |
| 14 | OMO372 | ATGGGATATTTTTGTCAATGAAGA | CAAGTGGCGTTAAGGTGTCAGTAG | (AT)9 | 304 | 60 |
| 15 | OMO051 | CAGCGGAGGAGGAGTCACAGG | AGTGGGAACAAGGGGATTAGACAT | (CT)18 | 278 | 60 |
| 15 | OMO069 | GGGCCGCCTCCTCCTTACAT | GCGGGTGGGAGGGTTGAACTA | (CA)16 | 194 | 60 |
| 15 | OMO074 | CAACTGTTTAAGGGCTTTGATGTT | AAGGCGACTTTTCTGAGATTGATA | (CA)10 | 429 | 55 |
| 15 | OMO099 | TTCCAGAATGGCAACAATAATGTA | AATAAATGTGTTCGCCACTGTAAT | (GT)15 | 234 | 60 |
| 15 | OMO379 | ATCCTCGCTGTCATCATCTCACTC | GAAACCAGCTTCACTGATAACAAA | (GT)13 | 395 | 60 |
| 15 | OMO409 | CTTGACTATGTAACGCATTCACTT | ATGATAGTAACGGCTGTTCCTGTG | (CA)8 | 334 | 55 |
| 15 | OMO416 | TCGGGAGGTGGAAGAACGATACA | CCACCCACCAAGTGAAGTGTGC | (GT)9 | 455 | 55 |
| 16 | GM581 | CAAAAGCAGCCACAAGTCAA | ACCTATGTGGGCTGAATGCT | (CA)34 | 150 | 55 |
| 16 | GM609 | GCACGGTCCTTACTGGTTGT | TCCCAGAGTGCCTTTGTTCT | (GT)22 | 106 | 55 |
| 16 | OMO130 | CTTGCTCTAGGATGATCTTCTGTG | TGAATGCAGCTGTGGTACAACTCC | (GT)11 | 140 | 55 |
| 16 | OMO366 | TGCTATGCTTGCTCAACCTTAGAT | ATGGCACTTGACAGAAACCTTTTA | (GT)20 | 271 | 60 |
| 16 | OMO378 | TTAATATTGGCACAAAAGTCTGGA | AGTCAACTGATGATGCACAAAAAG | (TA)11 | 337 | 60 |
| 16 | OMO397 | ACGCGTGTTTGAGATATTTAGATT | GAACAAACAAGGGGAGTGG | (GATT)7 | 222 | 60 |
| 16 | OMO407 | TGTGCAGCTTTCTTGGAGTTTCTA | TGGATTACTTAACGCAGCCTAACA | (GT)11 | 336 | 60 |
| 17 | GM342 | AAAGCCAAAACCAGCACA | TCAATCCTCTGTCACCCATA | (GT)13 | 264 | 55 |
| 17 | GM655 | AACCCTGTTGGCTGTCTCAC | CCGACAGTCAGCTCAGATCA | (CA)32 | 101 | 55 |
| 17 | OMO330 | TCAGAAGCAGCTTGAAAAATCA | TTTATTCCATGGTGAAAACATTCC | (GT)14 | 424 | 60 |
| 17 | OMO382 | CCATCCGCAGCTTCATTTTCATAC | AGCCAGAGCTGGAGGGACGATA | (CT)9 | 417 | 60 |
| 18 | OMO080 | CCAACCGACACATGTAACTCAAAA | AAACGGGTAGCTGCTGCTCTATGA | (GT)13 | 223 | 60 |
| 18 | OMO131 | TCCCTTAAAATATTACTGCTCCTC | AGTCCCATTTGCTTTACTACAGAC | (CA)13 | 139 | 55 |
| 18 | OMO236 | GGACCATGCCATCCATCT | CGCCACAGCGATCTGACTA | (GT)14 | 127 | 60 |
| 18 | OMO327 | TTATTTTTCAAACCCAAACTCAGC | CATGGGGTGTTATCAGATGTGC | (GT)12 | 272 | 55 |
| 18 | OMO341 | TGGAGCTCTACTTTGCCCCTACTA | ACGCTATAGATGGACCCTGGATTT | (CA)24 | 369 | 60 |
| 18 | OMO426 | ATGCGTGGTTATTAGGTGTGGTAT | TAATAGGATCGGTGACTTCAAACA | (AAGG)8 | 143 | 55 |
| 19 | GM112 | ATCCCGCTAACAGACTCA | CCGATATTCATCACGTTTAA | (CA)16 | 204 | 55 |
| 19 | GM175 | GGGGCTGTAAGTGTCTGTATG | GCAAACTGACCAAATGACC | (CA)27 | 192 | 55 |
| 19 | GM634 | CCCGACATTAAACTTTCAGCA | CTGAAACATGACTGCAGGAG | (GT)26 | 193 | 55 |
| 19 | OMO081 | ACAGCCCTGCTCCATTCATCTTTA | GCTCAGCCAGAGGGAAAAACACTA | (CA)8 | 457 | 55 |
| 19 | OMO210 | CCCCCAACACGCCTCAGAAG | GGTGCCAAAATAAGACCCAGTGC | (CA)9 | 150 | 55 |
| 19 | OMO281 | TCTTCACCGCTTGTATCTGTCACG | AAGGCTTGAAGCACATGAGTGGAT | (GT)25 | 293 | 60 |
| 19 | OMO310 | CAGTTAACTGGTAATTGCCTTTTG | ATTCTGCGTTGGTTTCTACTTTTT | (GA)13 | 151 | 60 |
| 20 | GM122 | AAAGGTCACAAAGGCAGCAAA | AGCCCCGCCCACAATAG | (CA)11 | 172 | 55 |
| 20 | OMO097 | TTTTTCCCCCACCGTTCTTCTCT | GCTGCTCTAACGCTGAACACAACC | (CA)13 | 224 | 60 |
| 20 | OMO129 | TTGGCAGGCTAAGTACTATTTCAT | GAGCGAATGGTTGTCTGTCTCT | (CCAT)9 | 267 | 55 |
| 20 | OMO253 | GGGTGGCGATCACATACTTTTCTA | CTCAAACAGTATGGTGGTGGATGG | (TA)8 | 243 | 55 |
| 20 | OMO288 | GTCCGGGCCCCTGACTCTAAC | GTCGTGCCAATGCTGTCACACTT | (GT)8 | 142 | 60 |
| 20 | OMO302 | TTTAGAGTCCTGGCTGGGTGTAAT | TGCTGTGTCACGGGACTCTTTTAG | (CT)16 | 318 | 55 |
| 20 | OMO360 | TGAATCCGGGCTAATTTACCTGTA | GTATTATTGGCCAACAACGCACAC | (CA)23 | 374 | 60 |
| 21 | OMO126 | GGTCGTAGCGATTTAGTCCAAACT | TTTTATGCGGCATGACAGATTTTA | (CT)15 | 173 | 60 |
| 21 | OMO209 | GCGGCACTTCTTTACATCTTACGA | AGGCGCAAAGCAGCATCAAA | (CA)27 | 247 | 55 |
| 21 | OMO247 | CTTGAAGGGAAGCGCTGTAAATAC | GCAGGAAGACGGCTGGGTGTA | (CA)11 | 432 | 55 |
| 21 | OMO301 | AGCCATTCCTCATTGATTGTCGTG | GGGGAGGGAGGGGGTTAGC | (CA)10 | 193 | 55 |
| 21 | OMO367 | CAGGCCAGAAAACAGCAAAACC | TGACCCTCATGTTCAGTTCAAAGA | (AAT)10(CA)11 | 346 | 60 |
| 21 | OMO375 | TGGCTAAATTTAGAGCTCACTGTT | GAAGGCAGGTAATCTAGCAAAAAG | (GT)11 | 367 | 60 |
| 21 | OMO392 | CTGGCTTAACTTCTCTACTGGACA | TCTACTCAAAACTGGCAACAAAAC | (GAATA)7 | 444 | 60 |
| 21 | OMO395 | AGATGCCTGAGCTGGAAAAGAGAT | TAAGCACCTGGATGGCATTATCAC | (CA)21 | 334 | 60 |
| 22 | OMO038 | ATACGCAGGAAATCTTCAAACACC | TTGCCCACTAGTGGTAAGAATCAA | (CAA)7 | 351 | 55 |
| 22 | OMO049 | AGCCAGCGGAGACCAACAAAAT | TCTCATGGGAGGCTCACTCACTCT | (GGAA)9 | 224 | 60 |
| 22 | OMO106 | CCGTTAAAGGAGCAGTCCAC | TTAGTGTTTCAATGCACAAATGAT | (CA)15 | 350 | 60 |
| 22 | OMO107 | AGCCCAAAACATCCCCTCTGTAA | CATGGGGTTTCTGTGACTCTTCCT | (CA)10 | 432 | 60 |
| 22 | OMO244 | TTTGCGTGTTCCCATCGTTTCTC | CTCCACATGCCGCCACAGC | (CT)10 | 434 | 55 |
| 22 | OMO433 | ATGTAGCGCTATCCACCTTCCATT | AAAAAGAGGCAGGCGAGTTCATAC | (CA)18 | 284 | 60 |
