# Supplementary material for: Mapping QTL for Sex and Growth Traits in Salt-Tolerant Tilapia (Oreochromis spp. X O. mossambicus)
Source: PLoS One. 2016 Nov 21;11(11):e0166723. doi: 10.1371/journal.pone.0166723 (PMC5117716; doi:10.1371/journal.pone.0166723)
Supplement: S2 Table — (DOCX) [file pone.0166723.s009.docx]

| **S2 Table : Phenotypic correlations coefficients between recorded traits of 180 dph fishes; BW – body weight, TL – total length, SL – standard length, BT – body thickness.** | | | | | |
| --- | --- | --- | --- | --- | --- |
|  |  |  |  |  |  |
|  | **180BW** | **180TL** | **180SL** | **180BT** |  |
| **180BW** | - | 0.93 | 0.88 | 0.7 |  |
| **180TL** |  | - | 0.95 | 0.69 |  |
| **180SL** |  |  | - | 0.72 |  |
| **180BT** |  |  |  | - |  |
