# Supplementary material for: Mapping QTL for Sex and Growth Traits in Salt-Tolerant Tilapia (Oreochromis spp. X O. mossambicus)
Source: PLoS One. 2016 Nov 21;11(11):e0166723. doi: 10.1371/journal.pone.0166723 (PMC5117716; doi:10.1371/journal.pone.0166723)
Supplement: S3 Table — (DOCX) [file pone.0166723.s010.docx]

|  | **S3 Table : Fine mapping marker pairs for male linkage group** | | | | |
| --- | --- | --- | --- | --- | --- |
|  |  |  |  |  |  |
| **Linkage** | **Marker name** | **Primer sequence (forward)** | **Primer sequence (reverse)** | **Product** | **Annealing** |
| **group** |  |  |  | **length (bp)** | **temperature (^o^C)** |
| 2 | 2RM22.3b | CTTTGACACAGTAGCGTATGATGC | TCGGAAATTTGACTGTGACCTACT | 368 | 55 |
|  | 2RM22.3a | CCTCCATAATGTAATAAAGTCCTC | AAAGTTACGCAGTAGTAGTGTTGA | 362 | 55 |
|  | 2RM20.6 | CATGACTGGGCCAGCTCCATCCTA | GAGGCGGTGATTTGGGGGTAATTG | 243 | 60 |
|  | 2RM20.4 | GGAAAGAAATGAAGGAAAACAAGA | GCCACCAGCATCACTCTCAC | 338 | 60 |
|  | 2RM19.0 | TCATCAGTAAAAGGGCTAATGTGT | CTCTAAGCTGGATAGTCCCTTTCT | 386 | 55 |
|  | 2RM17.6b | CGCCGTGGGAAGCTGGTAGGA | ACGGTGGTGAAGCGAAATGGAGTG | 401 | 60 |
| 18 | 18RM9.7b | GTGCCAGCGCTGCCACTCT | CTGTCAGAGGCGTAGAGGACGTTC | 285 | 60 |
|  | 18RM9.7a | AAAAACGGGGCCATGAGCATA | GGGCTGATGTAAGGCAGTCACAAG | 198 | 60 |
|  | 18RM21.6 | TGTCCGCCCTACAGGCTACATTC | AACAAGGAGTCGGGAGAAGGAACA | 264 | 55 |
|  | 18RM20.2 | AGCCAATTTAGCATCCCCTCAGGA | GATCACGTGGGCTGAAAGCGTATT | 408 | 55 |
|  | 18RM18.8 | CCAATGCGCTCTTCCACTTCTC | CAATTTGAGGCCGTTTGTATGGA | 359 | 55 |
|  | 18RM17.6b | GCCAATATTGCAGCAGGGTATCC | GAAAAAGAGCGGAAAACGCACAG | 357 | 60 |
|  | 18RM17.6a | GGGAATCTTCAGCGGCTGCTC | CCAGGGGTGACTGTGGGTCTCT | 234 | 60 |
|  | 18RM16.04 | AATATCGGGGATCCTCTCACCATT | GATGAAACAGCAAGGGAGGAGAGT | 390 | 60 |
|  | 18RM16 | GGTGTTTGGTTCCCCCTGAGA | TCAACCTCTACATCGGCATCTGG | 330 | 55 |
|  | 18RM13.8 | GTTGAGCGTGCATGTGTCTATGGA | GTGCTTGGCCACAGTGTTTCCTTA | 216 | 55 |
|  | 18RM10.4 | GCAGGGGTCAAAAAGCAATTAAGA | ACCAGCAGCAGCTTCATAACCATC | 331 | 60 |
|  | 18RM10.3 | GAAAATGGTCAAGCGGAAGG | AGGGCCGTGTCTGACTGCT | 284 | 60 |
|  |  |  |  |  |  |
