# Supplementary material for: Mapping QTL for Sex and Growth Traits in Salt-Tolerant Tilapia (Oreochromis spp. X O. mossambicus)
Source: PLoS One. 2016 Nov 21;11(11):e0166723. doi: 10.1371/journal.pone.0166723 (PMC5117716; doi:10.1371/journal.pone.0166723)
Supplement: S4 Table — (DOCX) [file pone.0166723.s011.docx]

| Table S4. Summary of genetic linkage map of all offsprings, male offsprings, female offsprings and male and female offsprings | | | | | | | | | | | | |
| --- | --- | --- | --- | --- | --- | --- | --- | --- | --- | --- | --- | --- |
|  |  |  |  |  |  |  |  |  |  |  |  |  |
| LG | Number of markers | | | | Length(cM) | | | | Average marker distance (cM) of individual linkage groups | | | |
|  | All | Male | Female | Male and female | All | Male | Female | Male and female | All | Male | Female | Male and female |
| 1 | 6 | 6 | 6 | 6 | 53.2 | 52.2 | 54.7 | 53.4 | 10.6 | 10.4 | 10.9 | 10.7 |
| 2 | 6 | 12 | 6 | 6 | 54.0 | 55.2 | 53.2 | 54.6 | 10.8 | 5.0 | 10.6 | 10.9 |
| 3 | 6 | 6 | 6 | 6 | 72.6 | 78.1 | 68.9 | 74.0 | 14.5 | 15.6 | 13.8 | 14.8 |
| 4 | 6 | 6 | 6 | 6 | 56.3 | 59.0 | 53.1 | 56.5 | 11.3 | 11.8 | 10.6 | 11.3 |
| 5 | 5 | 5 | 5 | 5 | 53.6 | 53.2 | 54.9 | 54.1 | 13.4 | 13.3 | 13.7 | 13.5 |
| 6 | 6 | 6 | 6 | 6 | 57.0 | 58.2 | 58.6 | 58.5 | 11.4 | 11.6 | 11.7 | 11.7 |
| 7 | 6 | 5 | 6 | 6 | 66.2 | 46.1 | 69.3 | 69.7 | 13.2 | 11.5 | 13.9 | 13.9 |
| 8 | 7 | 5 | 7 | 7 | 57.5 | 42.3 | 58.6 | 58.6 | 9.6 | 10.6 | 9.8 | 9.8 |
| 9 | 7 | 7 | 7 | 7 | 73.7 | 76.5 | 69.2 | 73.7 | 12.3 | 12.8 | 11.5 | 12.3 |
| 10 | 5 | 6 | 5 | 5 | 47.8 | 45.6 | 43.4 | 44.7 | 12.0 | 9.1 | 10.9 | 11.2 |
| 11 | 6 | 6 | 4 | 6 | 53.7 | 54.1 | 40.3 | 56.6 | 10.7 | 10.8 | 13.4 | 11.3 |
| 12 | 7 | 7 | 7 | 7 | 51.3 | 55.4 | 51.7 | 54.6 | 8.5 | 9.2 | 8.6 | 9.1 |
| 13 | 4 | 5 | 4 | 4 | 25.4 | 47.5 | 29.3 | 25.4 | 8.5 | 11.9 | 9.8 | 8.5 |
| 14 | 5 | 5 | 5 | 5 | 52.1 | 54.1 | 59.5 | 56.8 | 13.0 | 13.5 | 14.9 | 14.2 |
| 15 | 5 | 5 | 5 | 5 | 43.3 | 43.7 | 45.2 | 45.2 | 10.8 | 10.9 | 11.3 | 11.3 |
| 16 | 6 | 6 | 7 | 6 | 62.7 | 64.0 | 59.1 | 61.6 | 12.5 | 12.8 | 9.9 | 12.3 |
| 17 | 3 | 3 | 3 | 3 | 12.0 | 13.5 | 8.3 | 11.7 | 6.0 | 6.7 | 4.1 | 5.8 |
| 18 | 6 | 18 | 6 | 6 | 58.2 | 62.0 | 58.9 | 63.2 | 11.6 | 3.6 | 11.8 | 12.6 |
| 19 | 5 | 5 | 5 | 5 | 62.6 | 64.8 | 65.3 | 63.8 | 15.6 | 16.2 | 16.3 | 15.9 |
| 20 | 7 | 7 | 7 | 7 | 56.7 | 56.4 | 56.1 | 56.6 | 9.5 | 9.4 | 9.3 | 9.4 |
| 21 | 5 | 5 | 4 | 5 | 45.6 | 47.2 | 44.8 | 46.0 | 11.4 | 11.8 | 14.9 | 11.5 |
| 22 | 6 | 6 | 6 | 6 | 54.9 | 51.5 | 59.7 | 54.3 | 11.0 | 10.3 | 11.9 | 10.9 |
| Total | 125 | 142 | 123 | 125 | 1170.6 | 1180.8 | 1162.1 | 1193.5 | 11.4 | 9.8 | 11.5 | 15.6 |
|  |  |  |  |  |  |  |  |  |  |  |  |  |
