# Supplementary material for: Mapping QTL for Sex and Growth Traits in Salt-Tolerant Tilapia (Oreochromis spp. X O. mossambicus)
Source: PLoS One. 2016 Nov 21;11(11):e0166723. doi: 10.1371/journal.pone.0166723 (PMC5117716; doi:10.1371/journal.pone.0166723)
Supplement: S5 Table — (DOCX) [file pone.0166723.s012.docx]

| Table S5. Recombination ratios between male and female genetic maps | | | | | | |
| --- | --- | --- | --- | --- | --- | --- |
|  |  |  | |  | |  |
| LG | Number of shared markers | Length (cM) | | | | |
|  |  | Male | Female | | F:M | |
| 1 | 6 | 52.2 | 54.7 | | 1.0 | |
| 2 | 6 | 43.6 | 42.6 | | 1.0 | |
| 3 | 6 | 78.1 | 68.9 | | 0.9 | |
| 4 | 6 | 59.0 | 53.1 | | 0.9 | |
| 5 | 5 | 53.2 | 54.9 | | 1.0 | |
| 6 | 6 | 58.2 | 58.6 | | 1.0 | |
| 7 | 5 | 46.1 | 49.3 | | 1.1 | |
| 8 | 5 | 46.1 | 42.5 | | 0.9 | |
| 9 | 7 | 76.5 | 69.2 | | 0.9 | |
| 10 | 5 | 23.2 | 43.4 | | 1.9 | |
| 11 | 4 | 41.2 | 40.3 | | 1.0 | |
| 12 | 7 | 55.4 | 51.7 | | 0.9 | |
| 13 | 4 | 22.4 | 29.3 | | 1.3 | |
| 14 | 5 | 54.1 | 59.5 | | 1.1 | |
| 15 | 5 | 43.7 | 45.2 | | 1.0 | |
| 16 | 6 | 64.0 | 42.9 | | 0.7 | |
| 17 | 3 | 13.5 | 8.3 | | 0.6 | |
| 18 | 4 | 28.1 | 34.0 | | 1.2 | |
| 19 | 5 | 64.8 | 65.3 | | 1.0 | |
| 20 | 7 | 56.4 | 56.1 | | 1.0 | |
| 21 | 3 | 17.8 | 21.8 | | 1.2 | |
| 22 | 6 | 51.5 | 59.7 | | 1.2 | |
| Total | 116 | 1049.2 | 1051.3 | | 1.0 | |
